# Vehicles for Drug Delivery and Cosmetic Moisturizers: Review and Comparison

**DOI:** 10.3390/pharmaceutics13122012

**Published:** 2021-11-26

**Authors:** Tanya M. Barnes, Dalibor Mijaljica, Joshua P. Townley, Fabrizio Spada, Ian P. Harrison

**Affiliations:** Department of Scientific Affairs, Ego Pharmaceuticals Pty Ltd., Braeside, VIC 3195, Australia; tanya.barnes@optusnet.com.au (T.M.B.); Dalibor.Mijaljica@egopharm.com (D.M.); joshuat@egopharm.com (J.P.T.); fabrizio.spada@egopharm.com (F.S.)

**Keywords:** ointment, cream, gel, excipient, humectant, emollient, occludent, drug delivery, dermatitis, regulatory

## Abstract

Many dermatological conditions, such as eczema and psoriasis, are treated with topical therapeutic products. Instead of applying the active drug directly onto the skin, it is combined with a vehicle to aid in its delivery across the stratum corneum (SC) and into deeper regions of the skin, namely the epidermis and dermis. Absorption into the systemic circulation is minimized. Topical vehicles are also used as cosmetic moisturizers (often termed emollient therapy) to ameliorate dry skin, which is a cornerstone of the management of various dermatological conditions, including xerosis, eczema, psoriasis, and aging. The most common topical vehicles include ointments, creams, gels, and lotions, among others. It is crucial that topical vehicles are chosen based upon the size and properties (wet/dry, mucous/non-mucous, healthy/diseased) of the skin to be treated in order to optimize application and contact of the product with the skin, as this can have profound impacts on potency, efficacy, and patient compliance. This review examines common topical vehicles used for drug delivery and cosmetic moisturizers, including their formulation, advantages and disadvantages, and effects on the skin. The unique rules imposed by governing regulatory bodies in Australia and around the world, in terms of topical product claims, are also briefly examined.

## 1. Introduction

The skin is the outermost defensive barrier, which protects the human body from physical, chemical and microbial insults, and prevents the uncontrolled loss of water [1,2,3]. The barrier function of the skin resides in the stratum corneum (SC), which is composed of protein enriched corneocyte layers and intercellular membrane lipids, such as ceramides, cholesterol, and free fatty acids [1,2,3]. Corneocytes are rapidly and continually replaced to maintain skin hydration, flexibility and structural integrity, and repair any skin damage [1,2]. 

A dysfunctional skin barrier leads to skin dryness [4] and may be due to a genetic predisposition [5]; pathological causes such as eczema, psoriasis or xerosis [6,7]; environmental insults from the sun, wind or air conditioning [8,9]; frequent exposure to chemicals such as harsh soaps or detergents [10]; medications such as statins, diuretics or retinoids [11]; and other causes such as aging [12]. Dry skin exhibits a defective desquamation process, where corneocytes are shed as visible scales, causing a cosmetically unattractive rough texture and excessive transepidermal water loss (TEWL) [1,2,3]. Dry skin is unable to efficiently bind and hold water, and favors the penetration of irritants, allergens, and microorganisms, provoking discomfort and itchiness, as well as visible irritation or redness [1,2,3]. Thus, there is a need to protect both healthy and dry, compromised skin from a plethora of insults, and to preserve or restore its functional and structural integrity [3]. 

Topical vehicles are used as carrier systems, which aid in the delivery of an active drug (e.g., corticosteroid, calcineurins, vitamin D derivatives, retinoids) across the SC and into deeper regions of the skin, namely the epidermis and dermis, whilst minimizing absorption into the systemic circulation. These formulations are often used to treat dermatological conditions, such as eczema and psoriasis, to restore the skin barrier [2,3,6,7]. However, topical vehicles may also be used on their own as cosmetic moisturizers (often termed emollient therapy) to ameliorate dry skin, including conditions such as eczema and psoriasis where the use of moisturizers has become pivotal as first-line treatment strategies [2,7]. The most common topical vehicles include ointments, creams, gels, and lotions, among others. Topical vehicles are chosen based upon the nature (wet/dry, mucous/non-mucous, healthy/diseased) and size of the skin area to be treated to optimize application and contact of the product with the skin [13]. 

Prescribing effective topical therapies is crucial in the treatment of dermatological conditions as this can have profound impacts on their potency, efficacy, and patient compliance. Therefore, the aim of this review is to compare common topical vehicles used for drug delivery and cosmetic moisturizers according to their formulation, composition, intended use(s), effects on the skin, advantages and disadvantages, to provide a better understanding of their properties to enable the optimal choice for treatment. We also briefly examine the regulatory considerations for these products.

## 2. Topical Drug Delivery 

**Figure 1:**
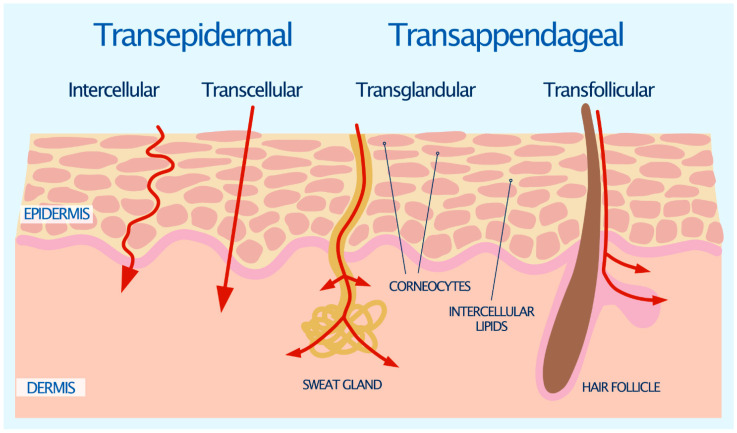
Drug permeation pathways through the stratum corneum [15].

The intact SC is considered to be the major barrier for drug penetration, as it is impermeable to almost all compounds and molecules with a molecular weight greater than 600 Daltons [14]. Diffusion along the concentration gradient is the principal mechanism by which the permeation of a drug across human skin takes place. There are two general pathways for drugs to permeate the SC: the transepidermal route and the transappendageal route [15], as illustrated in Figure 1.

### 2.1. Transepidermal Route

The transepidermal route contains two micropathways; the transcellular route and the intercellular route (Figure 1) [15]. The transcellular route is the most direct path for drugs to permeate the skin. It requires the drug to traverse the alternating layers of corneocytes and extracellular matrix, which is composed of an estimated 4–20 lipid lamellae [16]. This involves a sequence of partitioning and diffusion into alternating hydrophilic and lipophilic domains [15]. 

The more common pathway for drugs to permeate the skin is the intercellular route [17]. The intercellular route involves the drug diffusing around the corneocytes and through the continuous lipid matrix. The interdigitating nature of the corneocytes yields a tortuous pathway for intercellular drug permeation, which is in contrast to the relatively direct pathway of the transcellular route. It has been estimated that water has 50 times further to travel by the intercellular route than it does through the direct thickness of the SC [18]. Small hydrophilic drug molecules generally favor the transcellular route over the intercellular route, and vice versa for lipophilic molecules [19].

### 2.2. Transappendageal Route

The transappendageal, or shunt route, involves the flow of drug molecules through the sweat glands and hair follicles via the associated sebaceous glands (Figure 1). These skin appendages provide a continuous channel directly across the SC barrier. However, it is generally accepted that because the surface area occupied by sweat glands and hair follicles is small, typically only 0.1% of the skin’s total surface area, their contribution to epidermal permeation is also usually small [20]. Although sweat ducts provide a hydrophilic pathway across the skin due to the secretion of an aqueous salt solution, permeation may be limited as sweat moves in the reverse direction to that of the drug. In addition, sebaceous glands are filled with a lipid-rich sebum, which may present a barrier to hydrophilic drugs [21]. Even so, the transappendageal route can be vital for ions and large polar molecules which do not freely cross the SC [22].

## 3. The Role of the Vehicle

Delivering drug molecules to and through the skin involves the complex interplay between the active drug, the type of topical carrier system or ‘vehicle’, the choice of excipients, skin type and location, and skin condition. The topical carrier system, or ‘vehicle’, is defined as the substance that carries the chosen active drug into contact with and through the skin at an appropriate level to provide a therapeutic effect [23,24]. The challenge to topical drug delivery is the transport across the SC. To overcome this barrier, a vehicle must: maintain the solubility and stability of the active drug; release the active drug, depositing it on the skin with even distribution; enable penetration into and permeation through the SC skin barrier; facilitate partitioning from the SC into and diffusion through the viable epidermis; sustain the active drug at the target site for a sufficient duration to provide a therapeutic effect; and limit systemic absorption [23,25]. Furthermore, a vehicle should be soothing and comfortable, spread easily and be aesthetically pleasant, which aids in patient compliance [25]. These important attributes of a topical vehicle are dependent on the selection of the right excipients. 

## 4. Types of Vehicle

Topical vehicles can be classified based on their physical state, including semisolids (e.g., ointments, creams, gels) and liquids (e.g., lotions, solutions, foams, sprays) [24,26]. Lotions, gels and solutions are monophasic, ointments and creams are biphasic, whilst foams are triphasic [13]. Table 1 provides a summary of some common topical vehicles, suggested areas for application, and the advantages and disadvantages of their use.

### 4.1. Ointments

Ointment bases are classified into four general types, including: (1) hydrocarbon bases (e.g., petroleum, microcrystalline wax, ceresine); (2) absorption bases (e.g., wool fat, beeswax); (3) emulsion/water-removable bases (e.g., emulsifying wax); or (4) water-soluble bases (e.g., macrogols 200, 300, 400) [29]. Ointments (Table 1) are opaque or translucent, viscous, and have a greasy texture. They tend not to evaporate or be absorbed when rubbed onto the skin [28]. Ointments exert a strong emollient effect, softening, smoothing and lubricating the skin, which makes them useful in dry skin conditions. They form an occlusive layer over the skin, thus preventing the loss of water and heat [30]. This occlusive effect also enhances penetration of the active drug and improves efficacy, especially in thickened, lichenified skin [25]. Ointments are usually more difficult to spread compared to creams, lotions, and solutions [27]. Their greasy nature is cosmetically unappealing to some patients, which can limit compliance [31], particularly on hairy skin. Ointments are used most effectively in glabrous areas, namely the palms and soles, or on skin with short or sparse hair as they are difficult to wash off [32]. They are not suitable in wet, weepy dermatitis, hairy areas prone to folliculitis, or in hot weather due to their greasy and sticky nature, and their ability to retain sweat. Ointments require fewer preservatives than other vehicles since they contain little or no water [30].

### 4.2. Creams

Water-in-oil emulsions (oily creams) consist of water or an aqueous solution in the dispersed phase, and oil or oleaginous material in the continuous phase. These phases are reversed for oil-in-water emulsions (aqueous creams). Depending on the proportion of water and oil, creams range from mildly greasy to non-greasy. Water-soluble drugs are generally formulated in oil-in-water emulsions, while lipid soluble drugs are generally formulated in water-in-oil emulsions [33]. Creams (Table 1) are opaque and viscous, and tend to evaporate or be absorbed when rubbed onto the skin [28]. Creams do not retard heat loss as they are miscible with surface exudate. They are significantly less greasy, less viscous, and more spreadable than ointments, which makes them more appealing to some patients [27]. However, creams are comparatively less hydrating than ointments due to their inferior occlusive effect [27]. Creams are used for their moistening and emollient properties, making them effective in both dry and weepy/exudative skin conditions, especially those with significant exudate [27]. They are often chosen for infected and exudative plaques such as those found in eczema or psoriasis [30]. Creams can generally be used on all areas of the body including the face, and are especially effective in flexural and genital areas [30]. 

### 4.3. Gels 

Gels (Table 1) can be alcohol or water-based (hydrogel) and consist of transparent lattices of organic macromolecules, which are thickened with a gelling agent such as carboxymethylcellulose [33]. Gels tend to be thick and liquefy on contact with warm skin, providing a cooling sensation [28]. They dry to form a thin film which does not stain or leave behind a greasy texture [30]. These features make gels cosmetically favorable, however, they are poorly occlusive and generally do not provide hydration [27]. Gels are both easy to apply and wash off. They are particularly suitable for use in oily areas, such as the face, and also in hairy areas of the body [32]. As such, they are well suited for the treatment of acne and scalp conditions, such as seborrheic dermatitis and psoriasis [30]. A disadvantage of gels is that they are easily removed by perspiration [33], and alcohol-based gels may cause stinging if applied to inflamed or broken skin [29].

### 4.4. Lotions

Lotions (Table 1) generally contain an aqueous base in which a finely divided insoluble drug is suspended or dispersed [33]. They are opaque, thin, and have a non-greasy texture. Lotions are easily spread but only slightly occlusive, making them the least potent topical vehicle [33]. They are typically less moisturizing than creams. Lotions can be especially useful in the treatment of exudative dermatoses, as they can provide a cooling effect to the skin as the aqueous phase rapidly evaporates [28]. Lotions can be useful for hairy areas and are often used in the treatment of scalp conditions, such as seborrheic dermatitis and psoriasis [30]. In addition, they are also used on large areas due to their ease of application [33].

### 4.5. Solutions

Solutions (Table 1) are composed of one or more solutes dissolved in an aqueous, non-aqueous or hydroalcoholic solvent [27]. They may also contain a gelling agent to thicken the solution. Solutions have a clear appearance and a thin texture [28]. They are very simple to produce and easily spread over various areas of the body, including hairy areas such as the scalp [33]. Their liquid state makes them the most spreadable type of vehicle compared to creams and ointments [27]. However, solutions tend to be messy and provide no emolliating or skin protective properties, consequently providing only marginal skin hydration [27]. Further, due to the presence of alcohols, solutions are more prone to stinging when applied to inflamed or broken skin [26].

### 4.6. Foams

Foams (Table 1) are triphasic, consisting of oil, water, and organic solvent [34]. Although the matrix is stable at room temperature, it is thermolabile and breaks down rapidly, melting at approximately 32 °C, which is close to body temperature. At this point, the volatile constituents evaporate, resulting in minimal residue remaining on the skin [34]. Foams do not contain any fragrances, formaldehyde or non-formaldehyde preservatives [34]. They are cosmetically elegant making them easier to use in hairy areas, such as the scalp, as well as in oily areas, such as the face [32]. They are also effective in non-hairy areas [33]. Foams have the ability to penetrate the SC through the hair shaft [27]. They have important clinical implications in diseased states as they are generally less dense and easier to apply and spread on the skin surface. Specifically, in areas where the skin is overly inflamed or sensitive, foam application results in minimal mechanical sheering force to disperse the active drug [27]. However, depending on the foam base, application may result in stinging or burning to excoriated skin [33].

### 4.7. Sprays

Sprays (Table 1) exist as solution aerosols, consisting of a solution of the drug in a propellant (usually a blend of nonpolar hydrocarbons), or the propellant plus a suitable solvent such as ethanol, acetone, hexadecyl alcohol, glycol ethers or polyglycols [26]. Sprays can also be formulated as suspension aerosols where the drug is dispersed throughout both the propellant and solvent phase. However, suspension aerosols may exhibit problems including agglomeration, caking, particle-size growth, and valve clogging, which may be prevented by adding lubricants, surfactants or dispersing agents [35]. Sprays have the advantage of being able to treat large areas of affected skin, up to 15–20% body surface area [31]. Sprays are easily applied in a thin layer with little waste and good absorption [35], and are also useful for difficult to reach areas [36]. They may produce a cooling sensation upon application, however, they may also be associated with stinging and burning upon application [37]. In addition, there is no risk of contamination of the unused portion of the spray [35], making them an excellent choice of vehicle for the delivery of an active drug that need to be kept sterile, but also applied regularly. 

## 5. Choosing a Vehicle

Clinicians should undertake patient-specific assessments when choosing a suitable vehicle to deliver an active drug at the appropriate concentration to the skin, as the ultimate goal of a vehicle in this regard is to promote cutaneous absorption leading to enhanced clinical efficacy. The following vehicle attributes may be considered: the solubility, release rate and stability of the active drug in the vehicle; the ability of the vehicle to hydrate the SC; the physical and chemical interactions of the vehicle with the skin and the active drug; the anatomical localization and severity of disease; skin type; and patient preference [38]. In addition, the clinician should be aware of the hazards associated with a topical treatment, particularly the likelihood of irritant or allergic reactions [39]. 

Absorption of the active drug is influenced by skin properties that vary at different anatomical locations. For example, the absorption of an active drug diminishes greatly from the palpebral skin to the plantar surfaces [40]. Age also influences skin absorption, which is decreased in older individuals. Great variation is also noted for the skin of the premature infant and neonate, which have greater cutaneous permeability [41]. 

As a rule, acutely inflamed skin is best treated with fairly bland preparations that are least likely to irritate [38,39]. Moist or exudative eruptions are conventionally treated with ‘wet’ vehicles such as lotions or creams, whilst dry skin responds well to the occlusive action of ointments. Hair-bearing skin, especially the scalp, can be treated with lotions, gels or foams [38]. Palms and soles tend to respond well to occlusive ointments because of the thickness of the skin [38,39]. The cosmetic properties of the vehicle is of particular importance when treating the face. For example, oily skin affected by acne is often best treated with lotions or gels, whilst more sensitive skin affected by rosacea may benefit from the emollient effect of a cream [42]. Ointments, which generally do not contain preservatives or emulsifiers, can be an appropriate choice if allergy to these agents is a concern [30]. 

Cosmetic acceptability (greasiness, messiness, stickiness, visibility on skin, tactile sensation) and ease of use/convenience (spreadability, time required for application and drying, staining of clothes and bedding) is also important when deciding on a vehicle to satisfy patients, in order to increase compliance [42]. Poor adherence to topical medications has historically been a major factor behind the lack of treatment efficacy for many skin conditions [43,44]. Therefore, choosing the right vehicle is paramount to achieving success with a prescribed therapy, as well as avoiding wasted health care expenditure and patient disappointment [45,46]. Continual development and innovation of vehicle formulations are being designed to improve the aesthetic properties of topical therapies and patient satisfaction, adherence, and outcome [26].

In addition, the patient should be advised on the quantity of the topical therapeutic product to be used, where it should be applied, and the frequency and precise timing of application in relation to bathing and other treatments [39]. A realistic application quantity is rarely more than 5 mg/cm^2^. For semisolid vehicles, the fingertip unit (400–500 mg) has proven valuable [47]. For liquid vehicles, a mechanical dosage aid, such as a pump with weight data per pump stroke, can be used [39]. 

## 6. The Role of Moisturizers

Moisturizers are topical products specifically formulated to promote and maintain healthy skin and to keep it looking young and fresh. Hydrated skin is plump, luminescent and elastic, whereas dehydrated skin is tired looking, particularly around the eyes, dull and lacks spring and elasticity [11]. Moisturizers are also used to manage dry and itchy skin conditions in order to reduce the clinical signs of irritation and dryness (i.e., scaling and roughness), and to decrease the perceived feelings of tightness and itching [1,2,3]. Indeed, moisturizers often form the backbone of skin management strategies. For instance, the use of moisturizers (also known as emollient therapy) is a first-line treatment strategy alongside topical corticosteroids for the management of eczema [48].

Moisturizers improve skin hydration and increase SC water content by directly providing water to the skin from its water phase, and by increasing occlusion to reduce and prevent TEWL [49]. Furthermore, application of a moisturizer smooths the skin surface by filling spaces between partially desquamated skin flakes and restores the ability of the intercellular lipid bilayers to absorb, retain and redistribute water [5,11,25]. Skin mechanics change thereafter as increased hydration facilitates degradation of corneodesmosomes, preventing corneocyte accumulation while promoting its continuity [50].

Besides skin hydration, moisturizers can provide several other benefits to the skin. For instance, some moisturizers can inhibit the production of cytokines and pro-inflammatory prostanoids by blocking cyclooxygenase activity, thereby providing a soothing effect on inflamed skin, such as in eczema [51,52,53,54]. Moisturizers may also have anti-pruritic effects as they can downregulate various cytokines to reduce skin itchiness. Further, water-based moisturizers, as well as moisturizers containing methanol, can provide a cooling effect from evaporation on the skin surface, thereby reducing itch symptoms [52,53]. Moisturizers containing mineral oils have been shown to provide low-grade anti-mitotic actions on the epidermis and are thus useful in inflammatory dermatoses, such as psoriasis, where there is increased epidermal mitotic activity [52,53]. Moisturizers containing hyaluronic acid have been demonstrated to promote wound healing acceleration [54], while moisturizers containing sunscreen actives provide photoprotection [55]. Furthermore, some moisturizers exhibit anti-microbial activity against skin surface microbes [55]. Finally, moisturizer use may improve the quality of life, as smooth and hydrated skin plays a positive role in a patient’s social life and psychological satisfaction [55]. 

## 7. Types of Moisturizers

### 7.1. Primary Moisturizing Ingredients

**Figure 2:**
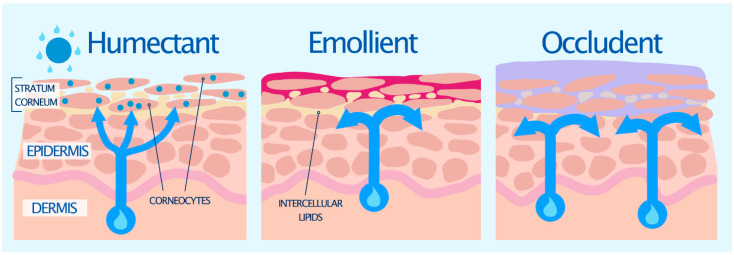
The mechanism of action of moisturizers [55,56].

Primary moisturizers can be divided into two types: lipophilic or hydrophilic. Hydrophilic moisturizers are also known as humectants, while lipophilic moisturizers can be subdivided into emollients and occludents [55,56]. Examples of each of these types of moisturizers are shown in Table 2, and their effects on the skin are illustrated in Figure 2. Often these ingredients are either the same or similar to natural components in the SC [57]. They are generally used in combination, with some ingredients providing an overlap of characteristics [56].

#### 7.1.1. Humectants

Humectants are hygroscopic substances that behave in a similar fashion to the natural moisturizing factor (NMF) in the skin [11,57]. Humectants readily penetrate the SC [58] and act like biological sponges by attracting and holding water in the skin, either by drawing it up from the dermis into the epidermis, or from the environment when the atmospheric humidity is >80% (Figure 2) [25,59]. They can also cause water to evaporate into the environment, and thus need to be used with occlusive agents to decrease or prevent TEWL, and help enhance epidermal barrier function and hydration [60]. Some humectants also possess emollient properties [61]. Many humectants are the same molecules that form the NMF, such as lactic acid, pyrrolidone carboxylic acid (PCA) and amino acids (Table 2) [11,57]. Humectants such as glycerol, triacetin, and polyols have traditionally been included into aqueous-based formulations, such as gels to improve the moisturizing and occlusive effect gels lack in comparison to creams and ointments [24]. 

#### 7.1.2. Emollients

Emollients simulate the intracellular bilayers of the SC [11,55,57]. They improve the ‘feel’ of the skin by filling the spaces in between corneocytes (Figure 2) and also provide what has been termed ‘skin slip’ or lubricity, imparting a sense of softness and plasticity [62]. This improves the overall appearance and texture of the skin. Some common emollients include essential fatty acids (e.g., linoleic acid, stearic acid, oleic acid, fatty alcohols) (Table 2), which are found in various natural oils (e.g., wool fat, palm oil, coconut oil). These essential fatty acids can be oxidized to eicosanoids, which are important signaling molecules involved in inflammatory pathways and the immune system. It is therefore thought that fatty acids may also influence skin physiology [61].

#### 7.1.3. Occludents

Occludents act like sebum and natural lipids found on the skin [11,57]. They reduce TEWL by forming a hydrophobic barrier film over the skin surface to prevent evaporation of water from the SC, trapping water in the skin’s uppermost layers (Figure 2) [63]. They have the most pronounced effect when applied to slightly dampened skin [60]. Common occludents include soft white paraffin/petrolatum, lanolin and waxes. Some lipophilic moisturizers, such as petrolatum, can enter the intercellular space of the SC and become part of the lipid structure to provide an increased barrier to water loss [58,64]. Through this interaction with the SC lipids, they effect an internal occlusion of the SC [58]. Petrolatum is often considered one of the most effective moisturizing ingredients for dry skin, which has been shown to reduce TEWL by more than 98% [65]. By contrast, other oily occludents including mineral oil, silicone, and lanolin (Table 2), reduce TEWL by about 20–30% [56]. The disadvantages of most occludents are their greasy feel, potential allergenicity, and odor [55].

### 7.2. Secondary Moisturizing Ingredients 

Besides the three primary types of moisturizing ingredients, many topical products also contain secondary moisturizers, those ingredients that offer additional hydrating benefits or enhance those offered by the primary group. The newest generation of moisturizers tend to contain these secondary ingredients to promote barrier repair. Notable examples of such ingredients are ceramides, free fatty acids, and cholesterol, which help replace the deficient lipids in some skin diseases characterized by barrier impairment, such as eczema and psoriasis [66,67]. 

Niacinamide is another secondary ingredient, which has been shown to reduce TEWL, strengthen the skin barrier, enhance the biosynthesis of ceramide and other lipids, prevent the transfer of melanosomes from melanocytes to keratinocytes, and possess an antiaging effect [68]. Other secondary ingredients include vitamins, such as vitamins C and E. These antioxidants protect cell membranes against oxidative stress and maintain the collagen network in the skin [69]. Further, botanicals such as *Avena sativa* (oat) kernel extract are also used as secondary ingredients. Oat avenanthramides are known to suppress histamine release at very low doses, helping to plump up the skin, reduce wrinkles, and restore the skin’s natural barrier [70].

## 8. Choosing a Moisturizer Vehicle

There are two important criteria for choosing a quality moisturizer. Firstly, it must contain a synergistic blend of moisturizing agents, including humectants, emollients, and occludents, which will help to improve efficacy. Secondly, it must be cosmetically acceptable; a good moisturizer is one that the patient will use [11]. As a general rule, the heavier the moisturizer the better the effect, but there is a need to balance the heaviness of a moisturizer with what the patient is willing to use [71]. Compliance is in accordance with patient preferences and desired results, hence, will likely be poor if the patient is unsatisfied with the moisturizer [51]. Ideally, clinicians should recommend therapeutic moisturizers that are non-comedogenic, non-irritating, and compatible with current therapeutic regimens [72].

The choice of moisturizer vehicle is generally related to the body area to be treated, the condition of the skin, and the patient’s preference [73]. Moisturizers are formulated for different body areas including the face, body, and hands or feet. Facial moisturizers are predominately oil-in-water emulsions (lotions) which can be recognized by their cool feel and nonglossy appearance. Water-in-oil emulsions (creams) can be recognized by their warm feel and glossy appearance [74]. Facial moisturizers are generally composed of vegetable/mineral oil or dimethicone, propylene glycol, glycerin, and water in sufficient quantity to form a lotion or cream [73]. Facial moisturizers can be developed for every complexion type by adjusting the occlusivity of the primary moisturizing ingredient. Oily complexion moisturizers are generally oil-free, composed of water and dimethicone, and are non-comedogenic and hypoallergenic. Moisturizers designed for normal/combination skin contain predominantly water, vegetable/mineral oil or dimethicone, and propylene glycol with very small amounts of petrolatum. Dry skin moisturizers contain water, vegetable/mineral oil, propylene glycol, and petrolatum [73].

Body moisturizers come in a variety of formulations, including lotions, creams, and ointments [65]. Lotions are generally the most popular formulation. Body lotions are generally oil-in-water emulsions containing 10–15% oil, 5–10% humectant, and 75–85% water [73]. More specifically, they are composed of water, vegetable/mineral oil, propylene glycol, stearic acid, and petrolatum. Most also contain an emulsifier, such as triethanolamine stearate, which is also a surfactant. Humectants such as glycerin or sorbitol may be used. Other additives include vitamins A, D, and E, and soothing agents such as aloe or allantoin [73].

Hand creams are oil-in-water emulsions with 15–40% oil, 5–15% humectant, and 45–80% water [75]. The addition of silicone derivatives can render the hand cream water-resistant through 4–6 washings. Most hand creams are based on petrolatum, glycerin, waxes, and dimethicone [38].

## 9. The Role of Excipients

The formulation of topical vehicles involves a great deal of skill. No single approach is suited to all drugs and uses, and thus a bespoke approach is required based upon the physiochemical properties of the active drug [23,24], and the area of the skin to be treated [38]. Some common non-active ingredients, commonly termed ‘excipients’, used in the formulation of topical vehicles, their role and effect on the skin, are shown in Table 3. Excipients typically make up greater than 90% of a topical product [24]. By their physicochemical nature, different classes of topical excipients are specifically used for designated purposes to enhance the functionality of each formulation, as well as overcome some of its challenges. Formulators seek to leverage excipient functionalities to develop products with specific performance attributes, including but not limited to improving solubility to allow incorporation of the drug at the target concentration; controlling drug release and permeation; improving general aesthetics of the product to increase patient compliance; improving drug skin permeability and/or deposition; improving drug and vehicle stability; and preventing microbial growth and contamination [24].

### 9.1. Stiffening Agents 

Stiffening agents (Table 3) are the main structure-forming excipients in topical semisolid formulations, such as ointments and creams [25,29,59]. A number of natural and synthetic lipids and hydrocarbons work as stiffening agents including white soft paraffin/petrolatum, liquid paraffin, lanolin, beeswax, carnauba wax, cetyl alcohol, and isohexadecane. Topical formulations with a high lipid content, as found in ointments and creams, form a protective occlusive barrier on the skin, protect from harmful substances, and help to keep the skin hydrated [25]. Stiffening agents also act as emollients to smooth, soften, and lubricate the skin by preventing TEWL. Topical vehicles containing stiffening agents are often used for dry and inflammatory skin conditions, such as eczema and psoriasis [24,25]. 

### 9.2. Thickeners/Gelling Agents

Thickeners (Table 3) are important excipients that influence topical vehicle viscosity, skin retention, and drug penetration [25,59]. There are four groups of thickeners: (1) lipid thickeners; (2) naturally-derived thickeners; (3) mineral thickeners; and (4) synthetic thickeners. Lipid thickeners (e.g., cetyl alcohol, stearic acid, carnauba wax) are usually solid at room temperature, but can be liquefied and added to emulsions. They work by imparting their natural thickness to the vehicle. Naturally-derived thickeners (e.g., hydroxyethyl cellulose, guar gum, xanthan gum, gelatin) are polymers that absorb water, causing them to swell up and increase the viscosity of the vehicle. Mineral thickeners (e.g., magnesium aluminium silicate, silica, bentonite) are also natural, and like naturally derived thickeners they absorb water and oils to increase viscosity, but produce a different result to the final emulsion. The final group are synthetic thickeners (e.g., cetyl palmitate, ammonium acryloyldimethyltaurate). They are often used in lotions and creams. The most common synthetic thickener is carbomer, an acrylic acid polymer that is water-swellable and can be used to form clear gels [59].

### 9.3. Silicones

Silicones (Table 3) act as non-greasy occlusive to aid in moisture retention [25,73]. They can also function as emollients, filling in spaces between desquamating corneocytes, to create a smooth skin surface that patients desire [73]. Dimethicone and cyclomethicone are the two most common silicones used in topical vehicle formulations [73].

### 9.4. Humectants

Humectants are described in Section 7.1.1 above.

### 9.5. Emulsifiers/Solubilisers

Emulsifiers (Table 3) play a significant role in the stability of topical vehicles by keeping dissimilar substances (such as oil and water) from separating, thus producing a homogeneous mixture with an even texture [24,25,59]. Emulsifiers are typically amphiphilic, possessing polar (hydrophilic) and non-polar (lipophilic) parts, which imparts more or less solubility either in water or in oil [76]. Emulsifiers that are more soluble in water will generally form oil-in-water emulsions, while emulsifiers that are more soluble in oil will form water-in-oil emulsions [76]. Examples of emulsifiers include anionic and non-ionic surfactants, polysaccharides, and glycerides [24].

A number of different chemical and physical processes are involved in emulsification, including a reduction in the interfacial tension between the two phases, known as surface tension theory. The emulsifier may also create a film over one phase that forms globules, which repel each other. This repulsive force causes them to remain suspended in the dispersion medium and is known as repulsion theory [76]. In addition, emulsifiers such as hydrocolloids (e.g., acacia, tragacanth), as well as polyethylene glycol, glycerine, and other polymers (e.g., carboxymethyl cellulose), all increase the viscosity of the medium, which helps create and maintain the suspension of globules of dispersed phase [76]. 

### 9.6. Solvents

Solvents (Table 3) play multiple roles, including enhancing the solubility of the active drug and facilitating drug absorption into the skin [25,59]. For aqueous-based topical formulations, such as aqueous gels and oil-in-water emulsions, water is often the main drug solvent, although various water-miscible solvents, such as polyols (e.g., polyethylene glycol and propylene glycol) and alcohols (e.g., ethanol, isopropyl alcohol, benzyl alcohol), can be included to improve drug solubility. Solvents enhance drug absorption through several mechanisms. At the site of topical application, volatile solvents, such as water, alcohol, and propellants (used in foams and sprays) evaporate, leading to enhanced drug absorption due to increased concentration [25]. In the case of topical vehicles that are already saturated with drugs, incorporation of solvents with relatively high boiling points may help to keep the drug from precipitating over a long period of time at the site of application, facilitating the absorption process [77]. Solvents are also incorporated in vehicles to dissolve excipients, such as coloring agents, preservatives, and stabilizers [25].

### 9.7. Penetration Enhancers

To facilitate drug delivery into the skin, excipients that disrupt the structure of the SC, referred to as penetration enhancers (Table 3), are used. Penetration enhancers may act by one of three main mechanisms: (1) disrupt the highly ordered structure of the SC lipid; (2) interact with the SC lipid; or (3) improve partitioning of the drug into the SC [77]. Penetration enhancers by nature damage the SC barrier. Consequently, care must be taken in selecting and using penetration enhancers at the optimal concentration to avoid unwarranted systemic absorption of the drug and skin irritation [78].

Many penetration enhancers are also solvents (e.g., propylene glycol, transcutol), and can be used alone or in combination to help facilitate both the partitioning into and the passage of a drug through the SC. Propylene glycol is also thought to integrate into the hydrophilic regions of the packed SC lipids and increase the solubility of this domain for the drug [79]. However, at high concentrations (above 10%) propylene glycol can irritate the skin [23,80]. Azones and dimethyl sulfoxide (DMSO) are also known to disrupt the lipid domains and improve the partitioning of drugs into the SC. In contrast, long chain fatty acids (e.g., oleic acid, linoleic acid) insert between the hydrophobic lipid tails to increase the fluidity of the lamellar bilayers [81]. For example, amitriptyline, one of the topical pain medications formulated in combination with other drugs, was found to permeate 4 to 5-fold more in the presence of fatty acids such as oleic acid. Surfactants and detergents also act as penetration enhancers by solubilizing the SC lipids [82]. 

### 9.8. Chelating Agents

Chelating agents (Table 3) such as ethylene diamine tetraacetate are used to enhance stability of a topical vehicle by binding metal ions to minimize metal-catalyzed degradation [29,79]. They can also enhance the effect of preservatives, which extend shelf life [59].

### 9.9. Acidifying/Alkalising/Buffering Agent

Apart from oxidation, the stability of drugs can also be affected by pH. Thus, buffers (Table 3) are used to maintain pH of topical vehicles throughout their shelf life [17,25,59]. Besides drugs, the behavior of certain excipients can also be affected by pH. For example, most carbomers have a pKa of around 6 and as such require a certain pH to hydrate and form a gel [17]. Therefore, the viscosity of a gel formulation comprising of a carbomer may be affected if significant changes in pH occur during storage [24]. 

### 9.10. Antioxidants

Many drugs in aqueous solution are susceptible to oxidative degradation, which may be prevented by the addition of an antioxidant [24,25,29,59]. The use of antioxidants (Table 3) can sometimes be avoided by reducing the amount of oxygen dissolved in a solution or present in the container, especially for single-use or sterile products [17]. The inclusion of certain excipients in the topical vehicles such as fixed oils, fats, and diethyl ether-based compounds, such as Transcutol P, which may contain low level peroxides, can also accelerate drug oxidation and should be avoided for drugs prone to oxidation. Antioxidants are also occasionally included to inhibit rancidity in topical vehicles containing unsaturated oils and fats, which are common in emulsion-based formulations [17]. Examples of commonly used antioxidants in topical formulations include alkyl gallates, butylated hydroxyanisole, butylated hydroxytoluene, ascorbyl palmitate, sodium ascorbyl phosphate, and tocopherols, where most exhibit synergistic effects when used in combination or in the presence of metal chelators such as edetic acid [22,24].

### 9.11. Preservatives

Preservatives (Table 3) are usually included in topical vehicles containing water, such as aqueous gels and creams, to prevent contamination and growth of microorganisms [24,29,59]. In non-aqueous systems, such as ointments, it is uncommon to include antimicrobial preservatives since microorganisms, while they may survive, rarely proliferate under such conditions. A preservative should be active against a wide spectrum of microorganisms and its selection should be based on several factors such as compatibility with the formulation, toxicity, irritancy potential, and the site at which the vehicle is to be applied [24,29]. The concentration of preservative should also be taken into consideration since other excipients within the vehicle may have some antimicrobial activity. Examples of some commonly used preservatives include alcohols (e.g., benzyl alcohol, ethanol, phenoxyethanol), hydroxybenzoates (all salts), phenols (e.g., chlorocresol), and quaternary ammonium compounds (e.g., benzalkonium chloride, cetrimide) [22,24].

## 10. Regulatory Considerations

In Australia, the Therapeutic Goods Administration (TGA) is the authority responsible for regulating prescription topical therapeutic products. Therapeutic goods are defined as products that prevent, diagnose or treat diseases, or that affect the structure or functions of the human body [83]. Topical therapeutic products must use ingredients approved by the TGA and listed on the Australian Register of Therapeutic Goods (ARTG) [83]. Ingredients not listed on the ARTG are required to undergo full non-clinical testing, including reproduction and fertility testing, and carcinogenicity studies. 

In contrast, over-the-counter (OTC) cosmetics, such as moisturizers, are not subject to premarket review and approval by the TGA. A cosmetic is defined as a substance that is designed to be used on any external part of the human body, or inside the mouth, to change its odors, change its appearance, cleanse it, keep it in good condition, perfume it or protect it [83]. Manufacturers are responsible for substantiating the safety of their products, and for labelling products with complete and accurate information regarding a product’s ingredients [83]. Ingredients in cosmetic products are classified as industrial chemicals, and new cosmetic ingredients are subject to notification to the National Industrial Chemicals Notification Assessment Scheme (NICNAS) for assessment unless they qualify for an exemption [83]. Formulators can check the conditions or restrictions of chemicals, which are available for use in Australia, using the Australian Inventory of Chemical Substances (AICS) and the NICNAS Cosmetics Guidelines. 

The Food and Drug Administration (FDA) is the authority responsible for regulating topical therapeutic products in the US. The definition of a therapeutic good and cosmetic in the US and EU is similar to that in Australia [83]. Formulators in the US must use ingredients listed on the FDA Inactive Ingredients Database (IID) to develop topical therapeutic products, which, similar to the ARTG in Australia, is much more restricted compared to the ingredients used to develop cosmetics [84]. For example, since 1957 there have only been three different emulsifiers used in developing 81 prescription topical creams in the US. This is most likely due to the fact that the FDA requires new ingredients to undergo full non-clinical testing, including reproduction and fertility testing and two year carcinogenicity testing in two species, which is estimated to cost up to USD 25 million [84]. In contrast, emulsifiers used in OTC moisturizers in the same time period were not listed on the IID, suggesting that innovation in the topical treatment of skin occurs almost exclusively within the field of cosmetics rather than medical dermatology [84]. 

In the US, similar to Australia, manufacturers are responsible for substantiating the safety of their products, and for labelling products with complete and accurate information regarding a product’s ingredients [83]. However, the FDA has abundant regulatory and enforcement authority for cosmetics under the federal Food, Drug and Cosmetics Act [83]. Consumers benefit from having FDA as a strong watchdog for their health and safety, and industry benefits when consumers are confident with the cosmetic safety standards set by the Agency. The FDA monitors the safety of cosmetic products in a variety of ways including: the Voluntary Cosmetic Registration Program; inspection of manufacturing facilities; surveys of products, including periodic purchasing and analysis; Cosmetic Ingredient Review (CIR) expert panel; and reports from consumers and health care providers through MedWatch [83].

In the EU, there is no comprehensive list of ingredients that has been approved for use in topical therapeutic products. In order to establish precedence of use, it is necessary to review the drug catalogues in each EU country such as the Dictionnaire Vidal in France, Die Rote Liste in Germany or The Electronic Medicines Compendium in the UK [85]. 

Cosmetics legislation adopted in 2013 in the EU, and enforced by each EU country, requires that every cosmetic product placed on the market is safe to use [83]. The regulation is based on three principles: (1) safety of the raw materials and ingredients; (2) good manufacturing practices (GMP); and (3) invigilating of the cosmetic market. These principles translate into requirements for each cosmetic brand, including designation of a responsible person; preparation of a product information file including a safety assessment; GMP for cosmetics; complying with labeling and packaging requirements; and notification via the Cosmetic Products Notification Portal (CPNP) [83]. Thus, the EU sets a higher level of transparency for finished cosmetic products, prevents the sale of hazardous substances, and strengthens safety for consumers.

In all, regulations can present a significant hurdle to innovation within dermatology, especially for topical therapeutic products. What may be acceptable in one country may not be in another, which often limits what can be achieved, or deemed worthy of investment in research and development time and capital. As such, new or innovative vehicles for topical drug delivery or cosmetic applications tend to be few and far between, which highlights the need for clinicians to understand and consider the options available now and use them appropriately, to better suit individual patient needs.

## 11. Conclusions

Topical drug delivery is the pillar of dermatologic therapy, and is far more complicated than just mixing an active drug into any ‘old cream’ and hoping for the best. The ability of a topical vehicle to deliver the active drug depends on how well the drug partitions from the vehicle into the skin, yet the skin’s intrinsic function to counteract the penetration of foreign substances by creating a physical barrier from the external environment makes drug delivery extremely complex. Therefore, the design of vehicles has a profound effect on the bioavailability, and consequently the clinical efficacy, of the active drug. In addition, the choice of topical vehicle is a crucial decision that can significantly alter efficacy, outcomes, and patient compliance. Therefore, clinicians must possess a fundamental knowledge of the composition, intended use(s), effects on the skin, advantages and disadvantages of the available topical vehicles when prescribing effective therapies for the treatment of dermatological conditions, such as eczema and psoriasis. 

The same formulation technology used to improve topical drug delivery and efficacy is also used to enhance the efficacy of cosmetic moisturizers to ameliorate dry skin due to environmental insults, frequent exposure to harsh chemicals, use of certain medications, and aging. In addition, the use of moisturizers has become pivotal as a first-line treatment strategy for various dermatological conditions, such as eczema and psoriasis. A quality moisturizer should contain a blend of moisturizing ingredients, including humectants, emollients, and occludents, which provide functional skin benefits, such as making the skin smooth and soft, increasing skin hydration, and improving skin aesthetic characteristics. The type of moisturizer vehicle is generally related to the body area to be treated, condition of the skin, and patient preference. Moisturizers can appear deceptively simple; however, the choices made by clinicians and patients can have a major impact on skin heath, healing, and wellbeing. 

## Figures and Tables

**Table 1:** Common topical vehicles, suggested areas for application, and the advantages and disadvantages of their use [25,26,27,28].

Vehicle	Definition	Areas of Application	Advantages	Disadvantages
Ointment	A suspension or emulsion semisolid formulation that contains <20% water and volatiles and >50% of hydrocarbons, waxes, or polyethylene glycols	Glabrous areas such as palms and soles Skin with short or sparse hair Drier areas of the body, such as the trunk and extremities Thickened and lichenified skin	Occlusive effect increases skin hydration, penetration and efficacy of active drug Emollient properties soothe and soften the skin Water resistance increases contact time Fewer preservatives reduce the risk of sensitization	Greasy which is less cosmetically appealing Difficult to wash off More difficult to spread than creams, lotions and solutions
Cream	An emulsion semisolid formulation that contains >20% water and volatiles and/or <50% of hydrocarbons, waxes, or polyethylene glycols	Dry and weepy/exudative skin conditions, especially if significant exudate Infected and exudative plaques Flexural and genital areas Can be used on all areas of the body including face	Moistening properties Emollient properties soothe and soften the skin More spreadable and less greasy than ointments resulting in increased patient compliance	Less occlusive and hydrating than ointments which decreases percutaneous drug absorption
Gel	A semisolid formulation that contains a gelling agent to provide stiffness to a solution or colloidal dispersion	Hairy areas, such as the scalp Oily areas, such as the face	Dries as a greaseless non-occlusive film Cosmetically elegant Easy to apply and wash off, especially on hairy areas Non-greasy Provides cooling sensation	No occlusive effects and minimal skin hydration Not an emollient Perspiration removes the gel Alcohol-based gels may cause stinging
Lotion	An emulsion liquid formulation with >50% water and volatiles	Exudative dermatoses Hairy areas, such as the scalp	Provide cooling effect as the aqueous phase evaporates Easy to apply to hairy areas Spreads rapidly	Less occlusive and hydrating than ointments or creams
Solution	A clear, homogeneous liquid formulation	Hairy areas, including the scalp Non-hairy areas	Easy to spread over various areas of the body, including the scalp Very simple to produce	Messy application No occlusive effects and minimal skin hydration Not an emollient Alcohol-based products can cause stinging, dryness, or skin irritation
Foam	Multiphase suspension containing a propellant stored under pressure and forming a foam upon release that quickly breaks down on the skin	Hairy areas of the body, such as the scalp Oily areas, such as the face Non-hairy areas Inflamed or sensitive areas, as application requires minimal mechanical sheering force to disperse the medication	Do not contain any fragrances, formaldehyde, or non-formaldehyde preservatives Low density and easy to apply and spread on skin surface Increased skin absorption Low-residue cosmetic elegance	No occlusive effects and minimal skin hydration Depending on the vehicle base, may result in stinging or burning to excoriated skin
Spray	A solution formulation with pressurized propellant	Can be used on all skin areas, but caution in skin folds where absorption is higher	Treats large areas of affected skin (up to 15–20% body surface area) Easier to apply to difficult to reach areas Easily applied in a thin layer with little waste and good absorption Cooling sensation upon application No risk of contamination of the unused portion	May be associated with stinging and burning upon application

**Table 2:** Common moisturizers and their effects on the skin [49,55].

Moisturizer Type	Examples	Effects on the Skin
Humectants	Glycerin, gelatin, propylene glycol, butylene glycol, panthenol, sorbitol, urea, hyaluronic acid, glycolic acid, lactic acid, sodium pyrrolidine carboxylic acid	Mostly low molecular weight substances which attract and hold water in the skin May cause water to evaporate into the environment and therefore need to be used with an occludent
Emollients	Cholesterol, squalene, linoleic acid, stearic acid, oleic acid, fatty alcohols	Saturated and unsaturated variable length hydrocarbons which improve the ‘feel’ of the skin by filling the spaces in between corneocytes Provide what has been termed ‘skin slip’ or lubricity, imparting a sense of softness and plasticity to the skin Improve the overall appearance and texture of the skin Often used in combination with emulsifiers
Occludents	White soft paraffin/petrolatum, beeswax, mineral oil, dimethicone, lanolin, carnauba wax, cetyl alcohol, caprylic/capric triglyceride	Oils and waxes which form an inert layer on the skin and physically block transepidermal water loss (TEWL) Some occludents enter the intercellular space and interact with the stratum corneum lipids, reinforcing the skin barrier

Note that some examples have multiple properties and effects on the skin.

**Table 3:** Common excipients, their role in topical vehicle formulation and effects on the skin [22,25,29,59].

Excipient	Examples	Role in Topical Vehicle Formulation	Effects on the Skin
Stiffening agents (lipids and hydrocarbons)	White soft paraffin/petrolatum, liquid paraffin, lanolin, beeswax, carnauba wax, cetyl alcohol, isohexadecane,	Main structure forming materials for semisolid formulations	Occlusive and skin protecting Hydrating Soothing and softening
Thickening/ gelling agents	Carbomer, cetyl alcohol, stearic acid, carnauba wax, hydroxyethyl cellulose, guar gum, xanthan gum, gelatin, magnesium aluminium silicate, silica, bentonite, cetyl palmitate, ammonium acryloyldimethyltaurate	Main structure-forming ingredients for gels and viscosity-enhancing ingredients for creams and lotions	-
Silicones	Dimethicone, cyclomethicone	Lubricant and film-forming ingredient	Occlusive and skin protecting
Humectants (polyols)	Glycerol, sorbitol, propylene glycol, polyethylene glycol, 1,2,6-hexanetriol, triacetin	Promotes the retention of water in the vehicle and the skin	Moisturizing and skin protecting Skin barrier stabilizing
Emulsifiers/ solubilizers	Glycerol monostearate, cetostearyl alcohol, cetyl palmitate, sorbitan monostearate, polysorbate 20, polysorbate 80, polysorbate 60, poloxamer, emulsifying wax, sorbitan monooleate, sodium lauryl sulfate, propylene glycol monostearate, diethylene glycol monoethyl ether	Used to reduce the interfacial tension to stabilize emulsions and to improve the wetting and solubility of hydrophobic ingredients	Skin conditioning Harsh surfactants may have deleterious effects by dissolving lipids and irritating the skin
Solvents	Purified water, propylene glycol, hexylene glycol, oleyl alcohol, mineral oil/liquid paraffin, propylene carbonate	Used to dissolve or disperse the active drug	Various effects, as described for other excipients
Penetration enhancers	Propylene glycol, oleic acid, isopropyl myristate, ethanol, polyethylene glycol	Increases permeation by promoting the diffusion, partitioning, or the solubility of an active drug through the stratum corneum	Disrupts the skin barrier to enhance drug delivery May be irritating at high concentrations
Chelating agents	Ethylene diamine tetraacetate	Binds metal ions to minimize metal-catalyzed degradation and to enhance the preservative effect	-
Acidifying/alkalizing/ buffering agents	Citric acid, lactic acid, phosphoric acid, sodium hydroxide	Maintains optimum pH for drug delivery	Products with high buffering capacity can alter skin surface pH
Antioxidants	Butylated hydroxyanisole, butylated hydroxytoluene, tocopherol, ascorbyl palmitate, sodium ascorbyl phosphate	To minimize oxidative deterioration and help stabilize the active drug	May reduce barrier disruption and inflammation due to oxidative stress within the stratum corneum
Preservatives	Benzoic acid, propyl paraben, methyl paraben, imidurea, sorbic acid, potassium sorbate, benzalkonium chloride, phenyl mercuric acetate, chlorobutanol, phenoxyethanol	Prevents microbial growth and contamination of the formulation	Added to protect the formulation rather than to exert an antiseptic effect on the skin

Note that many excipients have multiple properties and effects on the skin.
